# Mental Health Conditions and Severe COVID-19 Outcomes after Hospitalization, United States

**DOI:** 10.3201/eid2807.212208

**Published:** 2022-07

**Authors:** Alain K. Koyama, Emilia H. Koumans, Kanta Sircar, Amy M. Lavery, Jean Y. Ko, Joy Hsu, Kayla N. Anderson, David A. Siegel

**Affiliations:** Centers for Disease Control and Prevention, Atlanta, Georgia, USA

**Keywords:** mental health, depression, anxiety, bipolar disorder, schizophrenia, COVID-19, coronavirus disease, SARS-CoV-2, severe acute respiratory syndrome coronavirus 2, viruses, respiratory infections, zoonoses, vaccine-preventable diseases, United States

## Abstract

Among 664,956 hospitalized COVID-19 patients during March 2020–July 2021 in the United States, select mental health conditions (i.e., anxiety, depression, bipolar, schizophrenia) were associated with increased risk for same-hospital readmission and longer length of stay. Anxiety was also associated with increased risk for intensive care unit admission, invasive mechanical ventilation, and death.

Persons with mental health conditions (MHCs) might be at higher risk for severe COVID-19 outcomes after hospitalization because of poor access to care and a higher incidence of underlying conditions. Most studies have been limited by small samples or aggregation of MHCs, which can conceal differences in risk (*1*,*2*). Previous studies also have not examined length of stay (LOS) and readmission as outcomes. We examined patient records from a large, US-based electronic database to determine whether select MHCs were associated with severe COVID-19 outcomes, increased LOS, and same-hospital readmission.

The Premier Healthcare Database Special COVID-19 Release (accessed October 1, 2021) contained discharge data from >900 hospitals, representing ≈20 of annual admissions in the United States. (*3*). We identified patients hospitalized with COVID-19 and discharged during March 1, 2020–July 31, 2021, by using discharge codes from the International Classification of Diseases, 10th Revision, Clinical Modification (B97.29 for March 2020–April 2020 or U07.1 for April 2020–July 2021). MHCs of interest were anxiety, depression, bipolar disorder, and schizophrenia (identified from encounters from January 2019 through the index COVID-19 admission). Because patients could have multiple MHC diagnoses, categories were not mutually exclusive. Outcomes were intensive care unit (ICU) admission, invasive mechanical ventilation (IMV), 30-day same-hospital readmission (all-cause), in-hospital death (all-cause), and LOS. We used mixed effects models to examine the association between each MHC and each outcome. The reference group comprised patients who did not have MHC diagnoses of any type (i.e., anxiety, depression, bipolar disorder, schizophrenia, attention-deficit/hyperactivity disorder, obsessive-compulsive disorder, severe stress and adjustment disorders, eating disorders, disruptive disorders, impulse-control disorders, and conduct disorders). 

We used logistic models to estimate adjusted odds ratios (aORs) and corresponding 95% CIs for each dichotomous outcome (ICU admission, IMV, readmission, and death) and Poisson models to estimate the percentage difference and 95% CIs for LOS. A random intercept accounted for clustering by hospitals. We adjusted models for age, sex, race and ethnicity, insurance type, admission month, hospital characteristics (urbanicity and US Census Division region), and the Elixhauser Comorbidity Index (a measure of overall comorbidity based on 29 conditions) (*4*). We used SAS 9.4 (SAS Institute, https://www.sas.com) for statistical analyses.

Of our study sample of 664,956 hospitalized patients, 77.1% of patients were >50 years of age (Table). Male patients outnumbered female patients in having no MHC diagnoses (55.0%) or schizophrenia (53.8%); female patients outnumbered male patients in having anxiety (61.0%), depression (61.7%), or bipolar disorder (58.8%). We stratified COVID-19 outcomes among hospitalized patients by MHC diagnosis (Figure). Patients with anxiety, compared with those without any MHC, had a significantly higher odds of ICU admission (aOR 1.36, 95% CI 1.34–1.38), IMV (aOR 1.44, 95% CI 1.41–1.47), and in-hospital death (aOR 1.31, 95% CI 1.28–1.34). Patients with any of the MHCs, compared with patients without any MHC, had a significantly higher odds of readmission (anxiety, aOR 1.31 [95% CI 1.28–1.35]; depression: aOR 1.36 [95% CI 1.33–1.40]; bipolar disorder, aOR 1.50 [95% CI 1.41–1.59]; schizophrenia, aOR 1.40 [95% CI 1.31–1.49]). Similarly, each MHC was significantly associated with a longer mean LOS (anxiety, 34.8 days [95% CI 34.5–35.1]; depression, 19.5 days [95% CI 19.2–19.8]; bipolar disorder, 20.6 days [95% CI 19.9–21.2]; schizophrenia, 25.6 days [95% CI 24.9–26.3]).

**Table:** Characteristics of 664,956 hospitalized COVID-19 patients, by mental health condition diagnosis, from the Premier Healthcare Database Special COVID-19 Release, United States, March 2020–July 2021*

Parameter	No. (%) patients				
	No mental health conditions	Anxiety	Depression	Bipolar disorder	Schizophrenia
Total	485,784 (73.1)	114,902 (17.3)	96,167 (14.5)	15,370 (2.3)	12,304 (1.9)
Patient characteristics					
Age group, y					
0–17	5,415 (1.1)	608 (0.5)	584 (0.6)	97 (0.6)	25 (0.2)
18–39	61,751 (12.7)	11,584 (10.1)	7,598 (7.9)	2,527 (16.4)	1,421 (11.5)
40–49	50,127 (10.3)	11,863 (10.3)	7,949 (8.3)	1,996 (13.0)	1,095 (8.9)
50–64	129,896 (26.7)	33,511 (29.2)	26,320 (27.4)	5,455 (35.5)	4,251 (34.5)
65–74	101,996 (21.0)	26,561 (23.1)	23,756 (24.7)	3,298 (21.5)	3,512 (28.5)
>75	136,599 (28.1)	30,775 (26.8)	29,960 (31.2)	1,997 (13.0)	2,000 (16.3)
Sex					
F	218,428 (45.0)	70,105 (61.0)	59,327 (61.7)	9,032 (58.8)	5,682 (46.2)
M	267,356 (55.0)	44,797 (39.0)	36,840 (38.3)	6,338 (41.2)	6,622 (53.8)
Race/ethnicity					
Non-Hispanic White	241,534 (49.7)	78,634 (68.4)	67,015 (69.7)	10,267 (66.8)	6,409 (52.1)
Non-Hispanic Black	93,192 (19.2)	14,482 (12.6)	12,736 (13.2)	2,743 (17.8)	3,644 (29.6)
Hispanic	94,476 (19.4)	13,783 (12.0)	9,955 (10.4)	1,348 (8.8)	1,158 (9.4)
Non-Hispanic Asian	13,989 (2.9)	1,446 (1.3)	1,054 (1.1)	122 (0.8)	192 (1.6)
Other or unknown†	42,593 (8.8)	6,557 (5.7)	5,407 (5.6)	890 (5.8)	901 (7.3)
Health insurance					
Medicare	236,378 (48.7)	65,874 (57.3)	61,486 (63.9)	8,787 (57.2)	8,264 (67.2)
Medicaid	71,593 (14.7)	15,960 (13.9)	12,591 (13.1)	3,908 (25.4)	2,969 (24.1)
Private	133,735 (27.5)	26,102 (22.7)	17,015 (17.7)	1,817 (11.8)	493 (4.0)
Other†	44,078 (9.1)	6,966 (6.1)	5,075 (5.3)	858 (5.6)	578 (4.7)
Admission month					
2020 Mar or earlier	16,773 (3.5)	2,350 (2.0)	1,981 (2.1)	344 (2.2)	481 (3.9)
2020 Apr–Jun	70,536 (14.5)	12,946 (11.3)	11,968 (12.4)	2,578 (16.8)	2,959 (24.0)
2020 Jul–Sep	72,514 (14.9)	15,462 (13.5)	12,696 (13.2)	2,090 (13.6)	1,749 (14.2)
2020 Oct–Dec	149,494 (30.8)	40,166 (35.0)	34,329 (35.7)	4,830 (31.4)	3,651 (29.7)
2021 Jan–Mar	118,438 (24.4)	29,742 (25.9)	24,432 (25.4)	3,686 (24.0)	2,440 (19.8)
2021 Apr–Jun	46,674 (9.6)	11,904 (10.4)	9,076 (9.4)	1,587 (10.3)	863 (7.0)
2021 Jul	11,355 (2.3)	2,332 (2.0)	1,685 (1.8)	255 (1.7)	161 (1.3)
Elixhauser Comorbidity Index‡	4.0 (9.2)	6.2 (10.6)	6.9 (10.8)	5.7 (9.7)	6.4 (9.6)
Hospital characteristics					
Urbanicity					
Urban	585,009 (88.0)	429,073 (88.3)	99,530 (86.6)	83,505 (86.8)	13,682 (89.0)
Rural	79,947 (12.0)	56,711 (11.7)	15,372 (13.4)	12,662 (13.2)	1,688 (11.0)
Region					
East North Central	8,534 (1.8)	3,738 (3.3)	3,193 (3.3)	487 (3.2)	427 (3.5)
East South Central	79,512 (16.4)	15,288 (13.3)	13,917 (14.5)	2,567 (16.7)	2,669 (21.7)
Middle Atlantic	73,667 (15.2)	21,736 (18.9)	18,361 (19.1)	3,052 (19.9)	2,236 (18.2)
Mountain	19,812 (4.1)	6,684 (5.8)	6,544 (6.8)	747 (4.9)	520 (4.2)
New England	133,540 (27.5)	30,544 (26.6)	23,226 (24.2)	3,778 (24.6)	2,965 (24.1)
Pacific	32,915 (6.8)	9,537 (8.3)	7,610 (7.9)	957 (6.2)	608 (4.9)
South Atlantic	64,156 (13.2)	13,504 (11.8)	11,708 (12.2)	1,753 (11.4)	1,290 (10.5)
West North Central	39,377 (8.1)	8,394 (7.3)	6,840 (7.1)	1,180 (7.7)	727 (5.9)
West South Central	34,271 (7.1)	5,477 (4.8)	4,768 (5.0)	849 (5.5)	862 (7.0)

*Values are no. (%) except as indicated. †Missing values, when present, are categorized in the other category. ‡Higher values suggest a higher degree of comorbidity. Expressed as mean (+SD).

**Figure:**
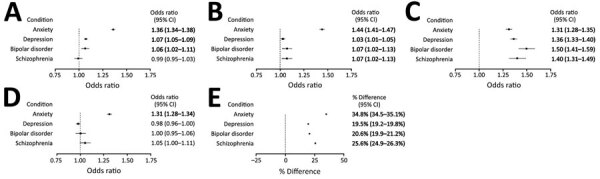
Outcomes of hospitalized COVID-19 patients (n = 664,956), by mental health condition diagnosis, compared with patients without mental health condition diagnoses in the Premier Healthcare Database Special COVID-19 Release, United States, March 2020–July 2021. For each condition, odds ratios represent the odds of the given outcome for patients with the condition compared with patients without mental health conditions. For length of stay, percentages represent the percentage difference in length of stay for patients with the condition compared with patients without mental health conditions. Covariates were selected based on factors known or plausibly associated with both the mental health condition and given outcome. Bolded values indicate statistical significance (2-sided α = 0.05), adjusted for multiple comparisons using the Bonferroni-Holm method. Descriptive statistics for each outcome, by mental health condition, and results from unadjusted models, are provided in the Appendix.

Anxiety was most strongly associated with severe outcomes in this patient sample; anxiety, depression, bipolar disorder, and schizophrenia were each independently associated with a higher risk of 30-day readmission and longer LOS. Comparing these results against the heterogeneous findings of prior studies is difficult for several reasons: aggregation of MHC, use of data early in the pandemic, populations with different risk profiles, and small samples (*2*,*5*,*6*). Most prior studies did not show a significant association between anxiety and a higher risk for ICU admission, IMV, or death (*2*,*5*), and most did not examine readmission or LOS as outcomes. MHCs might exacerbate respiratory disease and result in a greater risk for readmission or longer LOS in nonpsychiatric hospitalizations (*7*–*9*). These outcomes might be attributed to increased prevalence and severity of underlying conditions, immune dysregulation, use of psychotropic medications, socioeconomic disadvantage, or a combination of these factors (*8*,*9*).

Limitations of our study include residual confounding by such unavailable data as socioeconomic status, smoking status, and other substance use. MHCs among patients we studied might not have captured instances of milder disease because we identified those conditions by codes from the International Classification of Diseases, Tenth Revision, Clinical Modification. For example, the greater risk for death among patients with anxiety compared with patients with other MHCs could be attributed to differentially overcapturing more severe cases of anxiety. Hospital readmissions also might have been incompletely captured because data were only available on readmissions to the same hospital as the index admission for COVID-19. In addition, 58,743 patients (8.8%) had >1 MHC, potentially leading to misclassification.

By disaggregating MHCs, we demonstrated the differences in the risks associated with each individual condition. These findings might improve understanding of the risk for severe COVID-19 outcomes associated with MHCs and add evidence for considering MHCs as high-risk conditions for patients with COVID-19. 

Appendix Additional information about mental health conditions and severe COVID-19 outcomes after hospitalization, USA.
