# Supplementary material for: Mental Health Conditions and Severe COVID-19 Outcomes after Hospitalization, United States
Source: Emerg Infect Dis. 2022 Jul;28(7):1533–6. doi: 10.3201/eid2807.212208 (PMC9239877; doi:10.3201/eid2807.212208)
Supplement: Appendix — Additional information about mental health conditions and severe COVID-19 outcomes after hospitalization, USA. [file 21-2208-Techapp-s1.pdf]

Article DOI: http://doi.org/10.3201/eid2807.212208

Mental Health Conditions and Severe COVID-19 Outcomes after Hospitalization, United States

Appendix

Appendix Table 1. Outcomes of hospitalized COVID-19 patients, by mental health condition diagnosis, in the Premier Healthcare Database Special COVID-19 Release, March 2020-July 2021 (n = 664,956)

	Number of Cases, n (%)				
Outcome	Anxiety, n = 114,902	Depression, n = 96,167	Bipolar, n = 15,370	Schizophrenia, n = 12,304	No MHC, n = 485,784
ICU Admission	59,349 (51.7%)	45,943 (47.8%)	7,111 (46.3%)	5,811 (47.2%)	219,281 (45.1%)
Invasive Mechanical Ventilation	20,312 (17.7%)	13,841 (14.4%)	2,241 (14.6%)	2,104 (17.1%)	63,940 (13.2%)
30-Day Readmission	9,840 (8.6%)	9,218 (9.6%)	1,527 (9.9%)	1,201 (9.8%)	29,724 (6.1%)
In-Hospital Mortality	17,873 (15.6%)	13,303 (13.8%)	1,720 (11.2%)	1,780 (14.5%)	61,375 (12.6%)
Length of Stay, days, mean (SD)	10.3 (12.3)	9.4 (11.6)	9.2 (11.7)	10.6 (13.3)	7.7 (9.0)

Abbreviations: ICU = intensive care unit, MHC = mental health condition, SD = standard deviation.

Appendix Table 2. Regression equations used for inferential analyses.

Outcome: ICU Admission, Invasive Mechanical Ventilation, 30-Day Readmission, In-Hospital Mortality (logistic regression)

Unadjusted Model: $ln\left[\frac{p_i}{1-p_i}\right] = \beta_0 + \beta_1 X_{1i} + \varepsilon_i$

Adjusted Model: $ln\left[\frac{p_i}{1-p_i}\right] = \beta_0 + \beta_1 X_{1i} + \beta_2 X_{2i} + \beta_3 X_{3i} + \beta_4 X_{4i} + \beta_5 X_{5i} + \beta_5 X_{5i} + \beta_6 X_{6i} + \beta_7 X_{7i} + \beta_8 X_{8i} + \beta_9 X_{9i} + \varepsilon_i$

Outcome: Length of Stay (Poisson regression)

Unadjusted Model: $ln(\lambda_i) = \beta_0 + \beta_1 X_{1i} + \varepsilon_i$

Adjusted Model: $ln(\lambda_i) = \beta_0 + \beta_1 X_{1i} + \beta_2 X_{2i} + \beta_3 X_{3i} + \beta_4 X_{4i} + \beta_5 X_{5i} + \beta_5 X_{5i} + \beta_6 X_{6i} + \beta_7 X_{7i} + \beta_8 X_{8i} + \beta_9 X_{9i} + \varepsilon_i$

X_1 = mental health condition
X_2 = age
X_3 = sex
X_4 = race/ethnicity
X_5 = insurance type
X_6 = admission month
X_7 = urbanicity
X_8 = U.S. Census Division region
X_9 = Elixhauser Comorbidity Index

All regression analyses use mixed-effects models with a random intercept and variance components covariance structure, using the PROC GLIMMIX function in SAS 9.4.

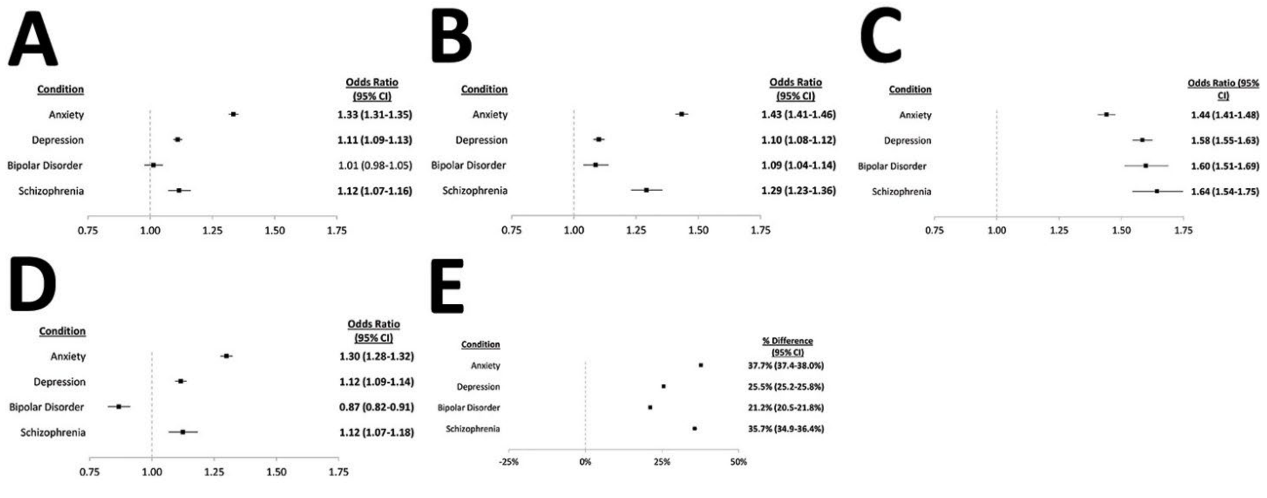

Appendix Figure. Unadjusted odds ratios for outcomes of hospitalized COVID-19 patients by mental health condition diagnosis compared to patients without any mental health condition diagnoses in the Premier Healthcare Database Special COVID-19 Release, March 2020-July 2021 (n = 664,956). For each condition, odds ratios represent the odds of the given outcome for patients with the condition compared to patients without any mental health conditions. For length of stay, percents represent the percent difference in length of stay for patients with the condition compared to patients without any mental health conditions. Bolded values indicate statistical significance (two-sided $\alpha = 0.05$), adjusted for multiple comparisons using the Bonferroni-Holm method.
